# Supplementary material for: Emerging technologies and research ethics: Developing editorial policy using a scoping review and reference panel
Source: PLoS One. 2024 Oct 31;19(10):e0309715. doi: 10.1371/journal.pone.0309715 (PMC11527293; doi:10.1371/journal.pone.0309715)
Supplement: S1 Fig — (DOCX) [file pone.0309715.s001.docx]

**Identification of studies via databases and registers**

Records removed *before screening*:

Duplicate records removed from Scopus records (n = 2)

Records identified from:

Google Scholar ^ (n = 269)

Scopus (n = 117)

Purposive sampling^#^ (n = 34)

COPE searches (n = 102)

**Identification**

Records excluded*

Google Scholar (n = 250)

Scopus (n = 43)

Purposive sampling (n = 0)

COPE (n = 0)

Records screened

Google Scholar (n = 269)

Scopus (n = 115)

Purposive sampling (n = 34)

COPE (n = 102)

**Screening**

Records excluded*

Google Scholar (n = 3)

Scopus (n = 6)

Purposive sampling (n = 0)

COPE (n = 63)

Record full-texts screened

Google Scholar (n = 19)

Scopus (n = 72)

Purposive sampling (n = 34)

COPE (n = 102)

Studies included in review

Google Scholar (n = 16)

Scopus (n = 66)

Purposive sampling (n = 34)

COPE (n = 33)

**Included**

The review process used both Google Scholar and Scopus in a serial fashion, and thus both sets of records are reported here through the whole process rather than merging after the initial searches; Searches conducted January 2023, Google Scholar searches were for terms indicating wider editorial policy, and Scopus searches specifically targeted ‘instructions to authors’. Full terms and detail regarding screening are provided in the supplementary materials (***S6 File. Supplement 6).***
