# Supplementary material for: Emerging technologies and research ethics: Developing editorial policy using a scoping review and reference panel
Source: PLoS One. 2024 Oct 31;19(10):e0309715. doi: 10.1371/journal.pone.0309715 (PMC11527293; doi:10.1371/journal.pone.0309715)
Supplement: S1 File — (DOCX) [file pone.0309715.s002.docx]

# Supplement 1: Joint Statement on Editorial Policy to Foster Learning Regarding Research Ethics

## Document Context

Supplement provides Joint Statement on Editorial Policy to Foster Learning Regarding Research Ethics.

## Document body

This joint statement provides a set of principles underpinning editorial policies to support learning about research ethics. Overall, editorial policy regarding research ethics should:

1. Foster ethical practice and expression within our research communities
2. Support learning about ethical practice and concepts across the communities that engage with us as researchers.

This statement of policy principles posits that:

1. **Research Ethics is grounded in:**
   1. Ensuring that research is conducted with consideration to respect, autonomy, and beneficence to participants in the research, and the wider impacts of our research in society;
   2. Conducting research with integrity (i.e., researcher conduct) and merit (i.e., suitable expertise and methods chosen and reported) is central to research ethics; research that is designed or reported in such a way that it cannot achieve its intended impact may unnecessarily use participant resources, present opportunity costs, and reduce trust in the integrity of the research endeavour;
   3. Respect for the contextual features of research sites and implementation, including disciplinary and societal norms and values. These may be negotiated over time and setting, with potential for norms to change, and for work that is deemed (un)acceptable in one context to be (un)acceptable in another, or to require different approaches to negotiation.
2. **Scholarly venues play a critical role in:**
   1. Advocating for and upholding the principles of ethical research;
   2. Providing a scholarly site for negotiating, and expressing, the ethical implications of research;
   3. Fostering awareness and supporting learning about ethical issues, across stakeholders, including the wider research community, and users of research evidence.
3. **Editorial policy plays a role in oversight and compliance with research ethics through:**
   1. A risk proportionate approach to ethical review as a component of compliance and oversight of harms, reflecting that this review is a key component of the ethics ecosystem;
   2. Requirements for authors that reflect the significance of ethical review, while not imposing unreasonable restrictions on work through, for example, acting as a secondary gatekeeper or requiring institutional approvals where these may not be locally available or required;
   3. At minimum, instructions for authors to follow the COPE “Responsible Research Publication: International Standards for Authors”. Even at this minimum level, statements that “ethics approval was granted by”, or “the study was exempt from”, should not be accepted as standalone statements. Explanations of these approvals or/and exemptions should be given, for both oversight reasons, and to support stakeholders to understand the ethics processes internationally, and their application to practical research.
4. **Editorial policy may support learning regarding ethics through:**
   1. *Encouraging* communities to consider expression of ethical concepts in their outputs, as not only an issue of compliance, but an opportunity to communicate to each other and to the public regarding research ethics;
   2. *Consideration of ethical resources* in terms of: the ethical concepts, values, and challenges faced in research; approaches to navigate dilemmas in research ethics; practical resources and tools used to address ethical issues; and nuances of ethical issues in specific contexts;
   3. *Resources* for authors, reviewers, and other stakeholders, provided both via venue/publisher websites, and in individual articles to support learning regarding ethical concepts. It may be appropriate for submission templates to make clear where ethics should/could be discussed, including formal reporting requirements, or through supplementary files*.*
   4. *Discussion* of the distinctive features of the area of research or application, stakeholder groups, settings, methods, and connection to disciplinary, cultural norms and values should receive particular attention. This may include issues such as dual-use research (research that may have applications that could cause harms through uses that are not the target of the research. For example, facial recognition), and long-range impacts (impacts that may be indirect, and on groups other than direct participants. For example, potential impacts of automated marking on labour relations);
   5. *Evaluation* of approaches to expressing and navigating ethical issues towards wider learning regarding them (for example, evaluation of editorial initiatives, considering appropriate publication pathways for work with ethics as a focus, spotlighting of key work).
