# Supplementary material for: Emerging technologies and research ethics: Developing editorial policy using a scoping review and reference panel
Source: PLoS One. 2024 Oct 31;19(10):e0309715. doi: 10.1371/journal.pone.0309715 (PMC11527293; doi:10.1371/journal.pone.0309715)
Supplement: S2 File — (DOCX) [file pone.0309715.s003.docx]

Supplement 2: Guidance for Authors on their Role in Fostering Learning Regarding Research Ethics

Document Context

Supplement provides Guidance for Authors on their Role in Fostering Learning Regarding Research Ethics.

Highlighted text indicates parts of the guidance where domain specific components may be adapted. In the example here we provide education-oriented domain specific guidance, to illustrate how tailoring to specific audiences may support learning regarding operationalisation of the guidelines.

Document body

Authors should include a statement regarding research ethics in all submissions. Where appropriate, this statement should note any institutional approval (including approval number), or a statement regarding the exemption of the research from such oversight, or rationale for approach where no committee is available. This statement should include a clear indication of participant consent process, and due consideration of issues of confidentiality and identifiability of participants. Further, authors must declare any potential conflicts of interest that might influence the research findings or interpretations. Reporting of these formal ethics issues supports transparency in the research process. It is the responsibility of authors to ensure compliance with relevant local policy regarding the ethical conduct of research.

Statements of research ethics also provide an opportunity to support learning in the research community and among the wider public to foster dialogue regarding ethical concepts and values underpinning research. For this reason, consideration of ethical issues should typically go beyond a simple “Ethics approval was granted by…”. Authors should consider how they can contribute to this learning through reflecting the ethical concepts drawn on, and situating their approach in any relevant literature. Ethical reflection may occur throughout a research project, from the selection of the topic and its problematisation, through its planning, conduct, and analysis, and dissemination for impact. It may be appropriate to include explicit articulation of this reflection in manuscripts, or/and manuscript supplementary files. In the domain of educational technology in particular, authors should reflect on issues of data privacy, with AI specifically on potential biases in the algorithms, and other issues such as the long-term impacts on student autonomy. Such reflections could guide educators and technology developers in making informed, ethically sound decisions.

1. The venue recognises that there is a balance in reporting results of the immediate work, and the benefits of conducting work that may have unanticipated outcomes and uses, alongside the desire to consider the potential for long-range impacts – both potential benefits and beneficiaries of research, and risks, harms, and to whom these accrue – where these may emerge after the work is conducted.
2. The venue is committed to research ethics and integrity in all matters; poor research has impacts not only for the individual participants and authorship teams, but for the wider practice of research and public trust in research. The venue follows COPE guidelines regarding issues of ethics and integrity.

The contribution that research makes to society is grounded in public trust in research, especially in humanistic fields like education and educational technology where the learner experience is central. Misrepresentation of research results, poor practice in research, and breaches of professional standards hamper this trust. For example, inaccurate portrayals of AI-driven educational interventions can skew perceptions and lead to ineffective educational strategies or policies. Conducting high quality research, and alignment of methods with specific questions and practical uses are all bound up in ethics. That is because at best the conduct of and participant involvement with low quality research or research that is not aligned to the issues it seeks to address (in research or society) is an opportunity cost, while at worst it may promote interventions that lead to poor outcomes. For example, such research may result in missed opportunities to engage learners in meaningful experiences, or at worst, lead to endorsement of educational technologies, promote practices or interventions that are ineffective or even lead to poor learning outcomes.

The integrity of research is supported by reporting, and fostering dialogue regarding:

- Issues of integrity and researcher conduct:
  - Allegations of misconduct and fraud will be investigated, where this is indicated it will be recorded
  - Disclosures should be made regarding the potential for any conflicts of interest
- Issues in the ethical conduct of research including reporting of:
  - Ethical oversight or approval of research
  - Consent processes for participants
  - Potential for reidentification in the data and any risk mitigation regarding issues of privacy
- Consideration of the impact of research
  - Long-range impacts including the potential benefits and harms that might accrue beyond individual participation in the research. While these impacts may be speculative and difficult to predict, a thoughtful consideration of risks and unintended consequences of moving research from development to deployment is crucial in promoting responsible and ethical research practices.
  - Secondary uses and potential for misuse must be clearly identified, and any limitations of outputs – including open data – for secondary purposes clearly highlighted
- Consideration of the merit or worth of research:
  - Transparency of data and methods, where appropriate within manuscripts, or on request to assure the validity or trustworthiness of the research and its findings
  - Appropriate representation of research results, which underpins integrity of the research endeavour and trust in it
  - Meeting standards of rigour appropriate to the discipline, domain of application, and specific questions being addressed.
