# Supplementary material for: Emerging technologies and research ethics: Developing editorial policy using a scoping review and reference panel
Source: PLoS One. 2024 Oct 31;19(10):e0309715. doi: 10.1371/journal.pone.0309715 (PMC11527293; doi:10.1371/journal.pone.0309715)
Supplement: S3 File — (DOCX) [file pone.0309715.s004.docx]

Supplement 3: Guidance for Reviewers on their Role in Fostering Learning Regarding Research Ethics

Document Context

Supplement provides Guidance for Reviewers on their Role in Fostering Learning Regarding Research Ethics.

Venues may wish to select questions based on submission type. All items are adapted from prior work (although their expression is original), except the final two items which were identified by the present researchers as missing in the checklists reviewed. Readers should review the published data file for further detail.

Venues should consider the most appropriate presentation of these prompts, and how guidance regarding ethical concerns can be integrated into wider reviewer material.

These prompts particularly target digital data and its computational analysis or use in automated decision making tools; they should serve as a model for the range of issues that may be considered in light of emerging technologies or novel contexts.

Document body

Reviewers play an important role in evaluating the appropriateness of an article’s discussion of ethical issues. Where relevant, this may include overseeing that research has ethics review and approval at a relevant institution, that consent has been provided for any data, and that steps have been taken to reduce risks of re-identification of participants and other harms. Reviewers should explicitly indicate if there are concerns with the research in this regard.

Reviewers should also consider the suitability of expression of ethical considerations, and whether ethical issues relevant to the work and the context of the study are clear, and would support constructive engagement with dialogue around ethical issues in research. Consider how the data in this paper were collected, analysed and reported. Can you suggest any way in which the authors might amend the paper to highlight how they addressed ethical aspects of this research?

The following questions may support this process.

| **Research Focus** | **Reviewer Prompt** | **Ethics Concept** |
| --- | --- | --- |
| **Data** | In obtaining, modelling, or sharing data have you considered the potential for bias? Bias may occur in the way people are represented, or in/excluded from datasets, or through the application of models to populations. and may occur ‘downstream’ as a result of data/models shared. Bias may also occur when using existing datasets or models. Consideration of any risks and their mitigation should be provided, drawing on relevant literature e.g., regarding existing models drawn on.  Where risks exist, do the burdens disproportionately impact already vulnerable populations? Do the benefits accrue equally to these populations? | bias |
| Data | Are the data and model adequately evaluated with respect to the issues (in research, or in a practical setting) addressed, noting that adequate evaluation is contingent on the claims being made and uses to which the work is put and limitations in this regard? | merit |
| Data | Has consent been provided for collecting and using the data? Are there risks of reidentification, and were these consented to? Where consent has not been sought, this should be explained. | consent |
| Data | Are there any risks to individuals or organisations such as professional harms due to the potential for reidentification of the data? | consent |
| Data | Is the intellectual property of others’ (including teachers and students) used in the research process, and on what grounds? This use may be based on your own collection of data (e.g., of student work), or the use of widely available datasets/models trained on ‘public’ data. | copyright |
| Data | Where identity characteristics may be relevant to your analysis, is their inclusion / exclusion motivated and adequately articulated? Do any identity characteristics perpetuate existing biases, or/and result in injustices in the burden and benefits of data collection or otherwise produce harms through categorisation of people? Do participants have opportunity to self-identify? Is it clear who any outcomes may work / not work for, and do these benefits / risks address issues of injustice? | justice |
| Data | Is it clear how personal data - particularly sensitive personal information – has been obtained, stored, processed, deidentified, and otherwise protected? | privacy |
| **Analysis** | If the paper uses crowd workers, student annotators, or other forms of low or no-compensation approaches to annotation or analysis, is there adequate description of the rationale for compensation and its fairness? And of the suitability of training for these workers? | labour law |
| Analysis | Where appropriate, is consideration clear regarding the potential for offensive content being encountered by either participants, researchers, or downstream users of research? (E.g., if learners may be exposed to misinformation, or employees to hate speech in labelling) | protection from harms |
| Analysis | Is there clear alignment between the questions being addressed and issues in research or a practical setting to which the work speaks, methods adopted, analytic results, and claims made? Is it clear where claims (of both potential harms and benefits) are more speculative, and where they are grounded in any results obtained and their potential for generalising? | merit |
| Analysis | Where possible are: datasets, qualitative codebooks, computational notebooks, models, and other materials made available, with a clear statement of license conditions (ideally open source or creative commons)? Is it clear what resourcing would be required to use the outcomes of the research in practice, or/and in further research projects? | merit |
| Implications | Have any of the research outputs been designed to foster informed human autonomy in tackling the potential for harms for both use of outputs as intended, and use where inaccurate or otherwise poor results are returned with intended use or misuses? | autonomy |
| Implications | Are possible positive and negative downstream impacts discussed? | downstream impact |
| Ethics | Is there a statement regarding ethics oversight by a relevant ethics committee or institutional review board, or if not, is there an explanation of the rationale for exemption, or the alternative approach adopted (e.g., where no ethics committee is available)? | oversight |
| Ethics | Have any power dynamics within and between researchers, participants, and other stakeholders been discussed? For example, this might include funders who are also participants or users or the research, or the power dynamics involved in educators conducting research in their own workplaces, or the dynamics between institutional or system-wide stakeholders. | power |
| Ethics | Have any considerations regarding vulnerable populations and appropriate gatekeepers been noted? For example, inclusion of children in research, and how their involvement in the research and consent process? | vulnerable Populations |
