# Supplementary material for: Emerging technologies and research ethics: Developing editorial policy using a scoping review and reference panel
Source: PLoS One. 2024 Oct 31;19(10):e0309715. doi: 10.1371/journal.pone.0309715 (PMC11527293; doi:10.1371/journal.pone.0309715)
Supplement: S4 File — (DOCX) [file pone.0309715.s005.docx]

Supplement 4: Guidance for Editors on their Role in Fostering Learning Regarding Research Ethics

Document Context

Supplement provides Guidance for Editors on their Role in Fostering Learning Regarding Research Ethics.

Venues may wish to focus their attention on particular topics, policy levers, or components of the publication process, as is appropriate to particular communities. The document sets out this possibility, and is intended to be adaptively used.

[Overview: Research Ethics and Learning 1](#_Toc169008824)

[Process for Evaluating Existing Policy 2](#_Toc169008825)

[Models for Embedding Research Ethics Editorial Flow 3](#_Toc169008826)

[Models for Editorial Oversight and Community Learning 3](#_Toc169008827)

[Other COPE resources 4](#_Toc169008828)

[References in file 6](#_Toc169008829)

Document body

Overview: Research Ethics and Learning

Editors play an important role in promoting research ethics and the positive impacts of research through their role in learned societies and publication venues (e.g., Abuhamad & Rheault, 2020; Ada Lovelace Institute et al., 2022; Prunkl et al., 2021). That is, both because of the significance of publication to most researchers, and the nature of editors as community leaders, with a position of overview across research being conducted and potential to influence the nature of that research (Ada Lovelace Institute, 2022). As the recent Cape Town Statement makes clear, venues also sit in a wider context of power imbalances in the costs and benefits of research and its dissemination, and play a role in addressing these inequities (Horn et al., 2023). Existing international policy requires published research involving human participation to include explicit statements regarding informed consent, and oversight or approval of an institutional ethics review (ICMJE, 2023) (Wager & Kleinert, 2010, 2011), with the COPE self-assessment for editors noting this as a core practice (COPE, 2022).

(COPE, 2022)The COPE ‘International standards for authors’ guidance, explicitly calls for reporting of ethics approval information, with a flowchart provided for cases where ethical concern is raised regarding a manuscript (COPE, 2021), with ethical oversight described as including: “but is not limited to, policies on consent to publication, publication on vulnerable populations, ethical conduct of research using animals, ethical conduct of research using human subjects, handling confidential data and ethical business/marketing practices.” (COPE, 2021). COPE provides detailed guidance in the document: “Responsible reporting of research involving humans or animals” (Wager & Kleinert, 2010, sec. 9).

This guidance document was developed to support editors in evaluating and developing policies and practices to foster ethics in research. It is intended to supplement the COPE ‘Ethics toolkit for a successful editorial office’ (COPE, 2022).

Process for Evaluating Existing Policy

Three key recent proposals have included discussion of policy foci and levers for fostering ethics in research publications, summarised in *Table 1*. Editors may wish to assess their existing materials against the six foci. #1 focuses on the editorial flow – the typical call, submission, review, and publication cycle; #2-6 are more focused on considerations relating to community dialogue.

*Table 1 Foci and Policy Levers for Fostering Ethics in Research Publications*

| **#** | **Strategy focus** | **Policy lever** |
| --- | --- | --- |
| 1 | Prescriptive and reflexive interventions to foster ethical reflection*;  e.g., “disclose and report additional information in their papers”^; “review potential downstream consequences earlier in the research pipeline”^  “expand peer review criteria to include engagement with potential downstream consequences and (6) establish separate review processes to evaluate papers based on risk and downstream consequences.”^ | **Instructions to:** authors, reviewers, and guest-editors/editorial-boards regarding the requirement to include particular issues.  **Submission template requirements** (published within article). This might include supplementary or structured elements, such as notes for practice (already adopted in some venues), or drawing on the range of resources available such as model cards or canvases of various kinds etc.  **Submission *form* requirements** (not published, or published as metadata). These include checkbox confirmations and fields included as part of the submission process. |
| 2 | training for reviewers and researchers*; | **Training provision** alongside instructions to authors, reviewers, etc., this might include: mentoring or author support particularly for junior authors; worked ‘cases’, examples of practice, or resources to benchmark author statements against; workshops/tutorials e.g., at conferences, covering aspects of the research-publication ecosystem and ethics, etc. |
| 3 | engagement with stakeholders impacted by tools*; | **Soft policy** to encourage research that engages with stakeholders in the design, development, evaluation, and implementation of tools. (see 1)  **Space within venues** for reflection on implementation or ethical engagement (see 4) |
| 4 | specifically drawing attention to work that exemplifies technical and ethical principles*;  “commend researchers who identify negative downstream consequences^;” | **Submission *categories* or types**, to spotlight or provide space for particular types of discussion within articles, or/and dialogue among articles.  **Awards** or other spotlighting mechanisms to highlight key work. |
| 5 | incentivise ‘slower’ research to support e.g. rolling submissions and R&R in conferences (rather than a one-shot speedy output)*; | **Policies to foster research**: Replicability (e.g. code/data sharing), Replications, and Replies (e.g. post-publication-review, commentaries re: implementation in practice, etc.) |
| 6 | Space for dialogue regarding the normative concerns of research.  (1) more research on the effects of ethics review processes+;  (2) more experimentation with such processes themselves+;  (3) the creation of venues in which diverse voices both within and beyond the AI or ML community can share insights and foster norms+;  “normalize discussion about the downstream consequences of research”^ | **Venues for dialogue:**   1. Materials to actively promote discussion including via editorials 2. Hosting workshops, round-table discussions, etc. on the topic 3. Provision of worked ‘cases’ 4. Resources to support consideration of both participant, and societal impacts over time |

Sources indicated by: ***(Ada Lovelace Institute et al., 2022)*; +*(Srikumar et al., 2022, p. 1061)*; ^*(Partnership on AI, 2021, p. 1)

Models for Embedding Research Ethics Editorial Flow

Submission forms should ensure that ethical concerns are addressed in the manuscript; if submissions are expected to follow structured templates in which ethical issues should be addressed in particular sections, it may be appropriate to simply use an overarching checkbox: “follows the template”. Consider whose role it is to assess whether this template has been adequately addressed.

Submission templates should identify space for:

1. Research Ethics Committee approvals, where appropriate, should be reported. Where none is available or required in the researcher's context, they should state this and why.
2. Wider ethical considerations should be discussed. Where this occurs will vary based on the manuscript, but it may include issues such as consent, long-range impacts, power dynamics, privacy, etc.
3. Ideally that supplementary files may be provided to discuss any key ethical issues and support wider addressing of integrity and merit concerns such as replicability.
4. Authors should explicitly reflect upon disclosures of interest and their impact on the nature of the work conducted.
5. An author contribution statement
6. Fields may wish to draw attention to particular sets of ethical principles (for example, where venues are published by a particular society, they may wish to highlight that community’s ethics materials)
7. Funding acknowledgements should be provided by the author/s.
8. Other acknowledgements.

If ‘ethics’ is provided as a section or heading in a template, consider how this might shape or limit the nature of coverage provided (see, e.g., discussion in Ashurst et al., 2021; Hecht et al., 2021).

Models for Editorial Oversight and Community Learning

Alongside the editorial process for individual submissions, editors may wish to consider the role of venues in fostering wider dialogue regarding research ethics. Editors may wish to develop approaches in consultation with their stakeholder communities (this may include research ‘users’) to foster community dialogue regarding the ethics of research.

Some policies are described in #2-6 above.

1. Spotlighting work: This may take the form of spotlighting quality (e.g. awards) regarding research on ethics, or research likely to foster ethics in research (e.g., through the transparency of approach, sharing of materials, etc.). It may also take the form of analysis beyond individual papers, perhaps of the longer-range impacts of works (in follow up assessment), or of the kinds of ethics issues highlighted across a set of works over a period.
2. Developing and engaging expertise in ethics: This may take the form of expert committees who can advise on ethics (e.g., The BMJ, 2022), or expert ‘ethics reviewers’ to augment the review process (e.g., NeurIPS, 2022), alongside support for events or resources regarding ethics.
3. Publication types: This may include editorials discussing key concerns or areas of focus, ensuring inclusion of space for consideration of ethics in all papers alongside space for submissions where this may be a particular focus. Editors may wish to consider how works relate to each other over time, and how this may be made visible for example, through ‘reply’ pieces, and evidence syntheses that reflect on the impact and ethical implications of research.

Editors may find it helpful to consult the COPE resources, including reported COPE cases of example issues and approaches to their resolution, and to consider sharing materials across communities.

Other COPE resources

COPE provide other materials to support navigating a range of issues, including:

- COPE provide “Guidance for Editors: Research, Audit and Service Evaluations” (COPE, 2014), a brief document covering the issue of what counts as ‘research’ in different countries for the purposes of ethical approval.
- COPE produce documents describing how journals should manage the specific method of medical case reports (highly identifiable) (Barbour et al., 2016);

COPE also reports cases, submitted by journals for discussion and advice. None are highly relevant to navigating nuanced issues around research ethics. A set of cases is provided below which may be useful for some purposes, although they are largely grounded in medical sciences.

*Re reidentification*

- Consideration of publishing raw data. (n.d.). COPE: Committee on Publication Ethics. Retrieved January 18, 2023, from <https://publicationethics.org/case/consideration-publishing-raw-data>
- Data anonymity. (n.d.). COPE: Committee on Publication Ethics. Retrieved January 18, 2023, from <https://publicationethics.org/case/data-anonymity>
- Ethics committee waives consent for case report, editor disagrees. (n.d.). COPE: Committee on Publication Ethics. Retrieved January 18, 2023, from <https://publicationethics.org/case/ethics-committee-waives-consent-case-report-editor-disagrees>
- Low risk study with no ethics committee approval. (n.d.). COPE: Committee on Publication Ethics. Retrieved January 18, 2023, from <https://publicationethics.org/case/low-risk-study-no-ethics-committee-approval>
- “Medical research” using data in the public domain. (n.d.). COPE: Committee on Publication Ethics. Retrieved January 18, 2023, from <https://publicationethics.org/case/%E2%80%9Cmedical-research%E2%80%9D-using-data-public-domain>
- Online trial of a new diagnostic tool. (n.d.). COPE: Committee on Publication Ethics. Retrieved January 18, 2023, from <https://publicationethics.org/case/online-trial-new-diagnostic-tool>
- Publishing complications and patient safety. (n.d.). COPE: Committee on Publication Ethics. Retrieved January 18, 2023, from <https://publicationethics.org/case/publishing-complications-and-patient-safety>

*Re: nature of research and research ethics approval*

- COPE Forum 8 July 2014: Ethical review. (n.d.). COPE: Committee on Publication Ethics. Retrieved January 18, 2023, from <https://publicationethics.org/resources/discussion-documents/cope-forum-8-july-2014-fair-play-%E2%80%9Cresearchers%E2%80%9D-can-editors-and>
- CV study: Was ethics approval and consent required? (n.d.). COPE: Committee on Publication Ethics. Retrieved January 18, 2023, from <https://publicationethics.org/case/cv-study-was-ethics-approval-and-consent-required>
- Ethical approval for a study. (n.d.). COPE: Committee on Publication Ethics. Retrieved January 18, 2023, from <https://publicationethics.org/case/ethical-approval-study>
- Ethical approval for retrospective study. (n.d.). COPE: Committee on Publication Ethics. Retrieved January 18, 2023, from <https://publicationethics.org/case/ethical-approval-retrospective-study>
- Ethics approval for audit 1. (n.d.). COPE: Committee on Publication Ethics. Retrieved January 18, 2023, from <https://publicationethics.org/case/ethics-approval-audit-1>
- Has formal ethical approval been granted that satisfies publication criteria? (n.d.). COPE: Committee on Publication Ethics. Retrieved January 18, 2023, from <https://publicationethics.org/case/has-formal-ethical-approval-been-granted-satisfies-publication-criteria>
- Inadequate assurance of human research ethics for a questionnaire. (n.d.). COPE: Committee on Publication Ethics. Retrieved January 18, 2023, from <https://publicationethics.org/case/inadequate-assurance-human-research-ethics-questionnaire>
- Is ethics approval required? (n.d.). COPE: Committee on Publication Ethics. Retrieved January 18, 2023, from <https://publicationethics.org/case/ethics-approval-required>
- No ethics committee approval of a study. (n.d.). COPE: Committee on Publication Ethics. Retrieved January 18, 2023, from <https://publicationethics.org/case/no-ethics-committee-approval-study>
- No ethics committee approval or informed consent. (n.d.). COPE: Committee on Publication Ethics. Retrieved January 18, 2023, from <https://publicationethics.org/case/no-ethics-committee-approval-or-informed-consent>
- Randomised controlled trial without ethics committee approval. (n.d.). COPE: Committee on Publication Ethics. Retrieved January 18, 2023, from <https://publicationethics.org/case/randomised-controlled-trial-without-ethics-committee-approval>
- Seeking retrospective ethics approval. (n.d.). COPE: Committee on Publication Ethics. Retrieved January 18, 2023, from <https://publicationethics.org/case/seeking-retrospective-ethics-approval>
- Service evaluation as research in a controversial area of medicine. (n.d.). COPE: Committee on Publication Ethics. Retrieved January 18, 2023, from <https://publicationethics.org/case/service-evaluation-research-controversial-area-medicine>
- The ethics of drug/medication use evaluation audit cycles and publication of the results. (n.d.). COPE: Committee on Publication Ethics. Retrieved January 18, 2023, from <https://publicationethics.org/case/ethics-drugmedication-use-evaluation-audit-cycles-and-publication-results>
- Was this study unethical? (n.d.). COPE: Committee on Publication Ethics. Retrieved January 18, 2023, from <https://publicationethics.org/case/was-study-unethical>
- Where should journals escalate serious concerns about an institution or institutional review board? (n.d.). COPE: Committee on Publication Ethics. Retrieved January 18, 2023, from <https://publicationethics.org/case/where-should-journals-escalate-serious-concerns-about-institution-or-institutional-review-board>

*Re: commercial sponsorship of research*

- Disagreement between authors and sponsor. (n.d.). COPE: Committee on Publication Ethics. Retrieved January 18, 2023, from <https://publicationethics.org/case/disagreement-between-authors-and-sponsor>

## References in file

Abuhamad, G., & Rheault, C. (2020). *Like a Researcher Stating Broader Impact For the Very First Time* (No. arXiv:2011.13032). arXiv. https://doi.org/10.48550/arXiv.2011.13032

Ada Lovelace Institute, CIFAR, & Partnership on AI. (2022). *A Culture of Ethical AI: Report*. CIFAR. https://partnershiponai.org/wp-content/uploads/dlm_uploads/2022/08/CIFAR-AI-Insights-EN-AM-220803-1.pdf

Ashurst, C., Hine, E., Sedille, P., & Carlier, A. (2021). *AI Ethics Statements—Analysis and lessons learnt from NeurIPS Broader Impact Statements* (No. arXiv:2111.01705). arXiv. https://doi.org/10.48550/arXiv.2111.01705

COPE. (2021). COPE Council. COPE Flowcharts and infographics Suspected ethical problem in a submitted manuscript—English. *COPE*. https://doi.org/10.24318/cope.2019.2.19

COPE. (2022). *Ethics toolkit for a successful editorial office*. Committee on Publication Ethics. https://doi.org/10.24318/AkFpEBd1

Hecht, B., Wilcox, L., Bigham, J. P., Schöning, J., Hoque, E., Ernst, J., Bisk, Y., De Russis, L., Yarosh, L., Anjum, B., Contractor, D., & Wu, C. (2021). *It’s Time to Do Something: Mitigating the Negative Impacts of Computing Through a Change to the Peer Review Process* (No. arXiv:2112.09544). arXiv. https://doi.org/10.48550/arXiv.2112.09544

Horn, L., Alba, S., Gopalakrishna, G., Kleinert, S., Kombe, F., Lavery, J. V., & Visagie, R. G. (2023). The Cape Town Statement on fairness, equity and diversity in research. *Nature*, *615*(7954), 790–793. https://doi.org/10.1038/d41586-023-00855-y

ICMJE. (2023). *Recommendations | Protection of Research Participants*. https://www.icmje.org/recommendations/browse/roles-and-responsibilities/protection-of-research-participants.html

NeurIPS. (2022). *NeurIPS 2022 Ethical Review Guidelines*.

Partnership on AI. (2021). *Managing the Risks of AI Research Six Recommendations for Responsible Publication*. Partnership on AI. https://partnershiponai.org/workstream/publication-norms-for-responsible-ai/

Prunkl, C. E. A., Ashurst, C., Anderljung, M., Webb, H., Leike, J., & Dafoe, A. (2021). Institutionalising Ethics in AI through Broader Impact Requirements. *Nature Machine Intelligence*, *3*(2), 104–110. https://doi.org/10.1038/s42256-021-00298-y

Srikumar, M., Finlay, R., Abuhamad, G., Ashurst, C., Campbell, R., Campbell-Ratcliffe, E., Hongo, H., Jordan, S. R., Lindley, J., & Ovadya, A. (2022). Advancing ethics review practices in AI research. *Nature Machine Intelligence*, 1–4.

The BMJ. (2022). *BMJ Knowledge Centre Committee on Ethics and Artificial Intelligence*. https://www.bmj.com/company/bmj-knowledge-centre-committee-on-ethics-and-artificial-intelligence/

Wager, E., & Kleinert, S. (2010). Responsible research publication: International standards for authors. *Promoting Research Integrity in a Global Environment. Singapore*, 309–316.

Wager, E., & Kleinert, S. (2011). Responsible Research Publication: International Standards for Editors. *PRILOZI*, *35*(3), 35–41. https://doi.org/10.1515/prilozi-2015-0006
