# Supplementary material for: Emerging technologies and research ethics: Developing editorial policy using a scoping review and reference panel
Source: PLoS One. 2024 Oct 31;19(10):e0309715. doi: 10.1371/journal.pone.0309715 (PMC11527293; doi:10.1371/journal.pone.0309715)
Supplement: S5 File — (DOCX) [file pone.0309715.s006.docx]

Supplement 5: Preferred Reporting Items for Systematic reviews and Meta-Analyses extension for Scoping Reviews (PRISMA-ScR)

Preferred Reporting Items for Systematic reviews and Meta-Analyses extension for Scoping Reviews (PRISMA-ScR), Checklist available from <http://www.prisma-statement.org/Extensions/ScopingReviews> and detailed in Tricco et al., (Tricco et al., 2018).

**PRISMA-ScR** **Checklist**

| **Section** | **Item** | **PRISMA-ScR** **checklist** **item** | **Reported page** **#** |
| --- | --- | --- | --- |
| **Title** |  |  |  |
| **Title** | 1 | Identify the report as a scoping review. | p.1 |
| **Abstract** |  |  |  |
| **Structured** **summary** | 2 | Provide a structured summary including, as applicable: background, objectives,  eligibility criteria, sources of evidence, charting methods, results and conclusions that relate to the review question(s) and objective(s). | p.1-2 |
| **Introduction** |  |  |  |
| **Rationale** | 3 | Describe the rationale for the review in the context of what is already known. Explain  why the review question(s)/objective(s) lend themselves to a scoping review approach. | p. 6 (§1.4 and 2.1) |
| **Objectives** | 4 | Provide an explicit statement of the question(s) and objective(s) being addressed with reference to their key elements (e.g., population or participants, concepts and context), or other relevant key elements used to conceptualize the review question(s)  and/or objective(s)). | p. 6 (§1.4 and 2.1) |
| **Methods** |  |  |  |
| **Protocol** **and** **registration** | 5 | Indicate if a review protocol exists, if and where it can be accessed (e.g., web  address), and, if available, provide registration information including registration number. | n/a (noted p. 6 §2.1) |
| **Eligibility**  **criteria** | 6 | Specify the characteristics of the sources of evidence (e.g., years considered,  language, publication status) used as criteria for eligibility, and provide a rationale. | p.7  §2.1.1 |
| **Information** **sources** | 7 | Describe all information sources (e.g., databases with dates of coverage, contact with  authors to identify additional sources) in the search, as well as the date the most recent search was executed. | p.7  §2.1.2 |
| **Search** | 8 | Present the full electronic search strategy for at least one database, including any  limits used, such that it could be repeated. | p.32  §Supplement 6.5 |
| **Selection** **of** **sources** **of**  **Evidence** | 9 | State the process for selecting sources of evidence (i.e., screening, eligibility) included  in the scoping review. | p.7  §2.1.1 |
| **Data charting process** | 10 | Describe the methods of charting data from the included sources of evidence (e.g., piloted forms; forms that have been tested by the team before their use, whether data charting was done independently, in duplicate) and any processes for obtaining  and confirming data from investigators. | p.8  §2.1.3 |
| **Data items** | 11 | List and define all variables for which data were sought and any assumptions and  simplifications made. |  |
| **Critical appraisal of individual sources of evidence** | 12 | If done, provide a rationale for conducting a critical appraisal of included sources of evidence; describe the methods used and how this information was used in any data synthesis (if appropriate). | n/a |
| **Synthesis of**  **results** | 13 | Describe the methods of handling and summarizing the data that were charted. | p.8  §2.1.3 |
| **Results** |  |  |  |
| **Selection of sources of**  **evidence** | 14 | Give numbers of sources of evidence screened, assessed for eligibility, and included in  the review, with reasons for exclusions at each stage, ideally using a flow diagram. | p.10 §3.1 |
| **Characteristics of**  **sources of evidence** | 15 | For each source of evidence, present characteristics for which data were charted and  provide the citations. | Supplementary data file |
| **Critical appraisal within sources of evidence** | 16 | If done, present data on critical appraisal of included sources of evidence (see item 12). | n/a |
| **Results of individual sources of evidence** | 17 | For each included source of evidence, present the relevant data that were charted that relate to the review question(s) and objective(s). | p.10 §3.1.1  and p.11 §3.1.2 |
| **Synthesis of results** | 18 | Summarize and/or present the charting results as they relate to the review question(s) and objective(s). | p.12  §3.2 |
| **Discussion** |  |  |  |
| **Summary** **of** **evidence** | 19 | Summarize the main results (including an overview of concepts, themes, and types of evidence available), explain how they relate to the review question(s) and objectives, and consider the relevance to key groups. | See item 20-21, and section 4 |
| **Limitations** | 20 | Discuss the limitations of the scoping review process. | p.14  §4.1 |
| **Conclusions** | 21 | Provide a general interpretation of the results with respect to the review question(s) and objective(s), as well as potential implications and/or next steps. | p.14  §4 |
| **Funding** |  |  |  |
| **Funding** | 22 | Describe sources of funding for the included sources of evidence, as well as sources of funding for the scoping review. Describe the role of the funders of the scoping review. | n/a (statement added in final publication by publisher) |

Tricco, A. C., Lillie, E., Zarin, W., O’Brien, K. K., Colquhoun, H., Levac, D., Moher, D., Peters, M. D. J., Horsley, T., Weeks, L., Hempel, S., Akl, E. A., Chang, C., McGowan, J., Stewart, L., Hartling, L., Aldcroft, A., Wilson, M. G., Garritty, C., … Straus, S. E. (2018). PRISMA Extension for Scoping Reviews (PRISMA-ScR): Checklist and Explanation. *Annals of Internal Medicine*, *169*(7), 467–473. https://doi.org/10.7326/M18-0850
