# Supplementary material for: Emerging technologies and research ethics: Developing editorial policy using a scoping review and reference panel
Source: PLoS One. 2024 Oct 31;19(10):e0309715. doi: 10.1371/journal.pone.0309715 (PMC11527293; doi:10.1371/journal.pone.0309715)
Supplement: S6 File — Modified PRISMA Flow Diagram of Scoping Review Search and Process. (DOCX) [file pone.0309715.s007.docx]

Supplement 6: PRISMA-Flow with Detailed Search Information

**Identification of studies via databases and registers**

Records removed *before screening*:

Duplicate records removed from Scopus records (n = 2)

Records identified from:

Google Scholar ^ (n = 269)

Scopus (n = 117)

Purposive sampling^#^ (n = 34)

COPE searches (n = 102)

**Identification**

Records excluded*

Google Scholar (n = 250)

Scopus (n = 43)

Purposive sampling (n = 0)

COPE (n = 0)

Records screened

Google Scholar (n = 269)

Scopus (n = 115)

Purposive sampling (n = 34)

COPE (n = 102)

**Screening**

Records excluded*

Google Scholar (n = 3)

Scopus (n = 6)

Purposive sampling (n = 0)

COPE (n = 63)

Record full-texts screened

Google Scholar (n = 19)

Scopus (n = 72)

Purposive sampling (n = 34)

COPE (n = 102)

Studies included in review

Google Scholar (n = 16)

Scopus (n = 66)

Purposive sampling (n = 34)

COPE (n = 33)

**Included**

The review process used both Google Scholar and Scopus in a serial fashion, and thus both sets of records are reported here through the whole process rather than merging after the initial searches; Searches conducted January 2023, Google Scholar searches were for terms indicating wider editorial policy, Scopus searches specifically targeted ‘instructions to authors’. Full terms and detail regarding screening are provided in the supplementary materials.

^ **Searches conducted January 2023.**

**Google Scholar search:** an initial search was conducted leading to n = 410 results:

("editorial policy" OR "editorial policies" OR "journal policies" OR "journal policy") ("ethics reporting" OR "ethical reporting" OR "ethics section")

On screening of Titles and snippets for relevance, a significant number of results appeared to focus on ethical *journalistic* reporting of sensitive topics. While the theme may be relevant for journals, the journalistic focus is likely out of scope. A second search excluding “journalistic” and “journalism” was conducted with 275 results reported. We recognise that this may have excluded some material of relevance from journalism disciplines. Note, while Scholar reported 275 results, only 269 were displayed.

("editorial policy" OR "editorial policies" OR "journal policies" OR "journal policy") ("ethics reporting" OR "ethical reporting" OR "ethics section") -journalistic -journalism

**Scopus search:** A Scopus query was constructed to identify analysis of author instructions, identified as a key research theme, limiting to documents: Article; Review; Editorial; Conference Paper; or Chapter.

TITLE-ABS-KEY ( ( *"instructions to authors"* OR *"instructions for authors"* ) AND ( *ethics* OR *ethical* ) ) AND ( LIMIT-TO ( DOCTYPE , *"ar"* ) OR LIMIT-TO ( DOCTYPE , *"re"* ) OR LIMIT-TO ( DOCTYPE , *"ed"* ) OR LIMIT-TO ( DOCTYPE , *"cp"* ) OR LIMIT-TO ( DOCTYPE , *"ch"* ) )

**Purposive sampling:** Was conducted through drawing on known examples of editorial policy addressing artificial intelligence and research ethics, including those cited in the key material drawn on in this paper.

**COPE searches:** Were conducted on the COPE website through manual navigation of their guidance resources with respect to research ethics and AI (excluding items relating to, e.g., publishing misconduct) particularly focusing on their pages on (ethical) oversight <https://publicationethics.org/oversight>. In addition, COPE cases were searched using the following query: “ethics committee”, with filter of topic set to “ethical oversight”

[https://publicationethics.org/search?t=%22ethics+committee%22&classification%5B%5D=2776&sort=score](https://publicationethics.org/search?t=%22ethics+committee%22&classification%5B%5D=2776&sort=score)](https://publicationethics.org/search?t=%22ethics+committee%22&classification%5B%5D=2776&sort=score%5d(https://publicationethics.org/search?t=%22ethics+committee%22&classification%5B%5D=2776&sort=score))

95 results were returned by this query.

* Screening was conducted on articles as follows, in the first instance using Title and Abstract fields (and in the case of Google Scholar, the search snippet), and for full-text review.

**Google Scholar screening:** Titles and search snippets were used for primary screening; abstracts were checked where further clarification was required. Items excluded for: (1) being about the ethics of an issue, where reporting is also discussed, but the theme is not about analysis of journal policies; or (2) journal policy discussion around other issues of academic integrity which we exclude here (e.g., conflicts of interest, or authorship standards).

**Scopus screening:** Article titles and abstracts were reviewed by 1 author, inclusion criteria were: A focus of the article was an analysis of either instructions to authors, or of published manuscripts, with respect to aspects of research ethics. Exclusions included: Article was an instruction to authors (some editorials with specific discussion of journal practices); abstract mentioned instructions to authors erroneously (e.g., it was copied in the metadata but should not have been), or research ethics was referred to but only with respect to an unrelated empirical work, or general guidance to authors (often novice authors) on publishing in particular venues.

**COPE screening:** COPE cases (n = 94) were screened for inclusion, with those excluded with n = 57 cases targeting misconduct, and n = 10 providing relatively clear cut or simple cases (e.g., authors claiming no research ethics was required when that was demonstrably false); n = 1 additional case discussed an example where approval seemed to have been provided but the researcher considered the work unethical, however the material provided was not deemed relevant to this review. Cases included (n = 26) focus on commercial interest (n = 1), whether the work constitutes research (n = 16), whether approval was sought (n = 2), and issues of reidentification (n = 7).

*Figure 1: Modified PRISMA Flow Diagram of Scoping Review Search and Process*
