# Supplementary material for: Emerging technologies and research ethics: Developing editorial policy using a scoping review and reference panel
Source: PLoS One. 2024 Oct 31;19(10):e0309715. doi: 10.1371/journal.pone.0309715 (PMC11527293; doi:10.1371/journal.pone.0309715)
Supplement: S7 File — Modified PRISMA Flow Diagram of Identification of Relevant Journals for Reference Group Invitation. (DOCX) [file pone.0309715.s008.docx]

Supplement 7: PRISMA-Flow of Journal Invites

**Identification of venues via SJR and purposive sampling**

Venues identified from SJR e-learning Q1+Q2 ranking (n = 72)

**Identification**

Records excluded n = 27 on initial screening titles irrelevant (e.g. “Government Information Quarterly”, or general education journals) or were not linked to a society.

A further n = 2 excluded, 1 re: development sector and 1 re: technology in library services

Sequential screening (i.e., if excluded in step 1, step 2 is not applied):

(1) Venue linked to scholarly society (n = 9)

(2) Link to learning and technology (n = 7)

**Screening**

N = 7 additional venues manually added

N = 7 venues identified representing n = 5 publishers

N = 9 publisher ethics boilerplate policies identified, alongside custom/self-published policies

**Included**

n = 2 venues did not reply to the invite or followup; n = 1 declined. Due to time constraints and practicalities of running reference groups no further invites were sent.

N = 6 initial venues invited representing publishers with >1 venues in results (3 major publishers + 2 OJS self-publishing venues)

N = 3 venues accepted invitation, representing two self-publishing / custom policies, and one customised major publisher policy

**Reference group**

*Figure 1: Modified PRISMA Flow Diagram of Identification of Relevant Journals for Reference Group Invitation*
