# Supplementary material for: Emerging technologies and research ethics: Developing editorial policy using a scoping review and reference panel
Source: PLoS One. 2024 Oct 31;19(10):e0309715. doi: 10.1371/journal.pone.0309715 (PMC11527293; doi:10.1371/journal.pone.0309715)
Supplement: S8 File — (DOCX) [file pone.0309715.s009.docx]

Supplement 8: ‘Policy Menu’ Examples

Examples of types of policy addressing research ethics:

- General COPE guidance (COPE, 2022)
- Submission templates (ACL et al., 2021; Ashurst et al., 2020; Beygelzimer et al., 2021; Hecht et al., 2021; NeurIPS, 2022)
- Indication of the significance of ethics in instructions to authors and reviewers, acceptance criteria, and review criteria (ACL et al., 2021; Ashurst et al., 2020; Beygelzimer et al., 2021; *Educationaldatamining.Org*, n.d.; Hecht et al., 2021; NeurIPS, 2022)
- Expert review by ethics-focused group (ACL et al., 2021; Ashurst et al., 2020; Beygelzimer et al., 2021; NeurIPS, 2022)
- Appraisal checklists or empirical standards including items regarding ethics (*Ethical Issues (Consent Etc.) | Report Sections | EQUATOR Network*, n.d.; *Supplements*, n.d.; Ralph et al., 2021; Simera, 2013) and post-publication appraisal checklist which includes “Is there evidence that the work has been approved by a specific, recognised committee? Are there any concerns about unethical practice?” (Grey et al., 2020).
- General statements of ethical principles (e.g., *ACM Code of Ethics Enforcement Procedures*, n.d.; *ACM FAccT - 2022 Disclosure of Funding Sources*, n.d.)

With some resources to foster learning regarding applying these:

- Networks for sharing resources and guidelines (*Ethical Issues (Consent Etc.) | Report Sections | EQUATOR Network*, n.d.; *Supplements*, n.d.; Ralph et al., 2021; Simera, 2013)
- Assessing impact and reflecting on this as a resource in fostering learning regarding positive and negative impacts of research, and enhance interaction with stakeholders (NeurIPS, 2022).

And further resources regarding fostering wider community dialogue:

- Resources that exemplify the application of principles within the particular domain (NIMEconference, 2020)
- Expert advisory group to provide advice regarding ethical issues (The BMJ, 2022)
- Engagement with researchers regarding approaches to foster ethics in their work (Holmes et al., 2021)
- There have been a number of workshops on the issue of research ethics (Fiesler, Bruckman, et al., 2018; Fiesler, Hancock, et al., 2018; Munteanu et al., 2019; e.g., Sturdee et al., 2021) COPE provides a forum, and case reporting, regarding ethics in publishing including research ethics (COPE, 2022)

## References in document

ACL, IJCNLP, & Rose, M. (2021). *ACL-IJCNLP Ethics Review and Ethics Review Questions*. ACL-IJCNLP 2021. https://2021.aclweb.org/ethics/Ethics-FAQ/

*ACM Code of Ethics Enforcement Procedures*. (n.d.). Retrieved February 27, 2023, from https://www.acm.org/code-of-ethics/enforcement-procedures

*ACM FAccT - 2022 Disclosure of Funding Sources*. (n.d.). Retrieved January 24, 2023, from https://facctconference.org/2022/funding_sources_disclosure.html

Ashurst, C., Anderljung, M., Prunkl, C. E. A., Leike, J., Gal, Y., Shevlane, T., & Dafoe, A. (2020, May 19). A Guide to Writing the NeurIPS Impact Statement. *Medium*. https://medium.com/@GovAI/a-guide-to-writing-the-neurips-impact-statement-4293b723f832

Beygelzimer, A., Dauphin, Y., Liang, P., & Wortman Vaughan, J. (2021, March 26). Introducing the NeurIPS 2021 Paper Checklist. *Medium*. https://neuripsconf.medium.com/introducing-the-neurips-2021-paper-checklist-3220d6df500b

COPE. (2022). *Ethics toolkit for a successful editorial office*. Committee on Publication Ethics. https://doi.org/10.24318/AkFpEBd1

*Educationaldatamining.org*. (n.d.). Retrieved April 13, 2023, from https://educationaldatamining.org/edm-2022-author-reviewer-checklist/

*Ethical issues (consent etc.) | Report Sections | EQUATOR Network*. (n.d.). Retrieved April 10, 2023, from https://www.equator-network.org/?post_type=eq_guidelines&eq_guidelines_study_design=0&eq_guidelines_clinical_specialty=0&eq_guidelines_report_section=ethical-issues-consent-etc&s=

Fiesler, C., Bruckman, A., Kraut, R. E., Muller, M., Munteanu, C., & Shilton, K. (2018). Research Ethics and Regulation: An Open Forum. *Companion of the 2018 Acm Conference on Computer Supported Cooperative Work and Social Computing (Cscw’18)*, 125–128. https://doi.org/10.1145/3272973.3274543

Fiesler, C., Hancock, J., Bruckman, A., Muller, M., Munteanu, C., & Densmore, M. (2018). Research Ethics for HCI: A Roundtable Discussion. *Chi 2018: Extended Abstracts of the 2018 Chi Conference on Human Factors in Computing Systems*, panel05. https://doi.org/10.1145/3170427.3186321

Grey, A., Bolland, M. J., Avenell, A., Klein, A. A., & Gunsalus, C. K. (2020). Check for publication integrity before misconduct. *Nature*, *577*(7789), 167–169. https://doi.org/10.1038/d41586-019-03959-6

Hecht, B., Wilcox, L., Bigham, J. P., Schöning, J., Hoque, E., Ernst, J., Bisk, Y., De Russis, L., Yarosh, L., Anjum, B., Contractor, D., & Wu, C. (2021). *It’s Time to Do Something: Mitigating the Negative Impacts of Computing Through a Change to the Peer Review Process* (No. arXiv:2112.09544). arXiv. https://doi.org/10.48550/arXiv.2112.09544

Holmes, W., Porayska-Pomsta, K., Holstein, K., Sutherland, E., Baker, T., Shum, S. B., Santos, O. C., Rodrigo, M. T., Cukurova, M., Bittencourt, I. I., & Koedinger, K. R. (2021). Ethics of AI in education: Towards a community-wide framework. *International Journal of Artificial Intelligence in Education*, *32*(3), 504–526. https://doi.org/10.1007/s40593-021-00239-1

Munteanu, C., Bruckman, A., Muller, M., Frauenberger, C., Fiesler, C., Kraut, R. E., Shilton, K., & Waycott, J. (2019). SIGCHI Research Ethics Town Hall. *Chi Ea ’19 Extended Abstracts: Extended Abstracts of the 2019 Chi Conference on Human Factors in Computing Systems*. https://doi.org/10.1145/3290607.3311742

NeurIPS. (2022). *NeurIPS 2022 Ethical Review Guidelines*.

NIMEconference. (2020, November 18). *NIME Principles & Code of Practice on Ethical Research*. NIME. https://nime.org/ethics/

Ralph, P., Ali, N. bin, Baltes, S., Bianculli, D., Diaz, J., Dittrich, Y., Ernst, N., Felderer, M., Feldt, R., Filieri, A., de França, B. B. N., Furia, C. A., Gay, G., Gold, N., Graziotin, D., He, P., Hoda, R., Juristo, N., Kitchenham, B., … Vegas, S. (2021). *Empirical Standards for Software Engineering Research* (No. arXiv:2010.03525). arXiv. https://doi.org/10.48550/arXiv.2010.03525

Simera, I. (2013). 5.6: Reporting guidelines: A tool to increase completeness, transparency, and value of health research published in your journal. In P. Smart, H. Maisonneuve, & A. Polderman (Eds.), *Science Editors’ Handbook European Association of Science Editors*.

Sturdee, M., Lindley, J., Linehan, C., Elsden, C., Kumar, N., Dillahunt, T., Mandryk, R., & Vines, J. (2021). Consequences, Schmonsequences! Considering the Future as Part of Publication and Peer Review in Computing Research. *Extended Abstracts of the 2021 CHI Conference on Human Factors in Computing Systems*, 1–4. https://doi.org/10.1145/3411763.3441330

*Supplements*. (n.d.). Empirical Standards. Retrieved March 14, 2023, from https://acmsigsoft.github.io/EmpiricalStandards/Supplements/

The BMJ. (2022). *BMJ Knowledge Centre Committee on Ethics and Artificial Intelligence*. https://www.bmj.com/company/bmj-knowledge-centre-committee-on-ethics-and-artificial-intelligence/
