# Supplementary material for: Emerging technologies and research ethics: Developing editorial policy using a scoping review and reference panel
Source: PLoS One. 2024 Oct 31;19(10):e0309715. doi: 10.1371/journal.pone.0309715 (PMC11527293; doi:10.1371/journal.pone.0309715)
Supplement: Attachment — Submitted filename: round_1_review.docx [file pone.0309715.s010.docx]

# Response to reviews letter

PONE-D-24-01120

Dear Dr Chi

Our thanks to the editors and reviewers for helpful comments in revising the submission.

**Publication statements**

I here submit the *revised* manuscript PONE-D-24-01120 to PLOS One. As lead author, I (Simon Knight) affirm that the paper has not been previously published in part or in whole, and is not under consideration in any other venue. We attach:

1. A response letter, responding to each point raised (below)
2. A marked-up copy of the manuscript, with changes tracked, labelled 'Revised Manuscript with Track Changes'.
3. An unmarked version of the revised paper without tracked changes. Labelled 'Manuscript'.

In addition to the response letter:

1. We update the Conflict of Interest Statement as requested. Advice is also sought which may have implications for this statement or/and the funding disclosure statement.
2. We confirm that we do not believe the lab protocol depositing is relevant to the work

**Response to reviews**

We appreciate the reviewers’ engagement with the content and substance of the submission. We’re pleased the reviewers note the originality and rigour of the research. We address all reviewer comments in the table below, and would particularly draw attention to points 11-12 regarding the role of AI technology with the manuscript and the corresponding coherence/connectedness of the sections, and point 17 regarding the disciplinary scope of the paper particularly with respect to education as an example sub-field. We believe these were two key areas for attention, and we hope that these responses address the points raised.

We hope the reviewers and editors find the responses and revisions suitable, and look forward to your further decision

Kind regards

Simon Knight (on behalf of the authors)

## **Revised Competing interests statement**

The research reported was instigated as a collaboration between the Journal of Learning Analytics (JLA), Australasian Journal of Educational Technology (AJET), and the British Journal of Educational Technology (BJET), by the lead author, who was a previous co-editor-in-chief of the JLA. The authors acknowledge BJET co-editor-in-chief Cathy Lewin, who joined the journal later in this process, and thus did not participate in authoring this piece.

The views expressed are those of the authors, and may not represent the views of the journals, scholarly societies, or other organisations to which they are affiliated. 
The work was begun over the course of the lead author’s sabbatical, which included periods co-located at UCL and KTH / the Swedish Digital Futures research centre, supporting direct interaction with editors based at those institutions.

During the write up of this research, the lead author received funding from the Australian Government through the Australian Research Council (ARC) Discovery Early Career Award (DECRA) Fellowship (DE230100065), and Discovery Project (DP, DP240100602). The views expressed herein are those of the authors and are not necessarily those of the Australian Government or Australian Research Council.

This does not alter our adherence to PLOS ONE policies on sharing data and materials.

# Detailed response to reviews

| **#** | **R** | **Comment** | **Reply** |
| --- | --- | --- | --- |
| 1 | E | 1. Please ensure that your manuscript meets PLOS ONE's style requirements, including those for file naming. The PLOS ONE style templates can be found at  <https://journals.plos.org/plosone/s/file?id=wjVg/PLOSOne_formatting_sample_main_body.pdf> and  <https://journals.plos.org/plosone/s/file?id=ba62/PLOSOne_formatting_sample_title_authors_affiliations.pdf> | Regarding file naming, see #5-9  Regarding style, our understanding is that PLOS does not require submissions to be formatted until they are provisionally accepted. Nevertheless, we have:   1. Reviewed the referencing format amending it to the PLOS variant of Vancouver style 2. Per #5-9 checked cross-referencing of supplementary files 3. Reviewed Tables, we believe these are correctly formatted (Title at top, caption/legend and footnotes below). 4. Reviewed figures, and extracted these to separate files, including the caption within the manuscript 5. Headings have been amended:    a. Level 1 is: Bold 18 sentence case    b. Level 2 is: Bold 16 sentence case    c. Level 3 is: Bold 14 sentence case   While the style template does not include numbered headings, we note the journal does use these, and thus have retained them in the manuscript (where we use them to cross-reference sections).   6. Paragraph indented 7. We have not yet amended to double space (for readability reasons) |
| 2 | E | Thank you for stating the following in the Competing Interests section:  "The research reported was instigated as a collaboration between the Journal of Learning Analytics (JLA), Australasian Journal of Educational Technology (AJET), and the British Journal of Educational Technology (BJET), by the lead author, who was a previous co-editor-in-chief of the JLA. The authors acknowledge BJET co-editor-in-chief Cathy Lewin, who joined the journal later in this process, and thus did not participate in authoring this piece. The views expressed are those of the authors, and may not represent the views of the journals, scholarly societies, or other organisations to which they are affiliated.  The work was begun over the course of the lead author’s sabbatical, which included periods co-located at UCL and KTH / the Swedish Digital Futures research centre, supporting direct interaction with editors based at those institutions."  Please confirm that this does not alter your adherence to all PLOS ONE policies on sharing data and materials, by including the following statement: ""This does not alter our adherence to PLOS ONE policies on sharing data and materials.” (as detailed online in our guide for authors <http://journals.plos.org/plosone/s/competing-interests>).  If there are restrictions on sharing of data and/or materials, please state these. Please note that we cannot proceed with consideration of your article until this information has been declared.  Please include your updated Competing Interests statement in your cover letter; we will change the online submission form on your behalf. | See subheading above.  I have also added information regarding funding received by the lead author. This funding was not specific to the research (the bulk of the research occurred prior to the funding starting) but was received while the manuscript was in development and under review. The PLOS policy states that this does not need to be declared, and so I believe the funding statement (“The author(s) received no specific funding for this work.”) remains appropriate. However, in the interests of transparency and recognition of the funding that I should mention it here or/and the acknowledgements. |
| 3 | E | Please note that in order to use the direct billing option the corresponding author must be affiliated with the chosen institute. Please either amend your manuscript to change the affiliation or corresponding author, or email us at [plosone@plos.org](mailto:plosone@plos.org) with a request to remove this option. | I liaised with UCL regarding this and they confirmed that in this instance they were happy to make an exception with respect to the PLOS agreement and honorary status (I have attached evidence of this).  I note this is not part of the review process. |
| 4 | E | When completing the data availability statement of the submission form, you indicated that you will make your data available on acceptance. We strongly recommend all authors decide on a data sharing plan before acceptance, as the process can be lengthy and hold up publication timelines. Please note that, though access restrictions are acceptable now, your entire data will need to be made freely accessible if your manuscript is accepted for publication. This policy applies to all data except where public deposition would breach compliance with the protocol approved by your research ethics board. If you are unable to adhere to our open data policy, please kindly revise your statement to explain your reasoning and we will seek the editor's input on an exemption. Please be assured that, once you have provided your new statement, the assessment of your exemption will not hold up the peer review process. | We said   - “Relevant data are contained within the manuscript and its Supporting Information files, except where data cannot be shared publicly due to copyright restrictions. In these cases, links to retrieve the original source data will be provided.”, and - ticked the box “Tick here if the URLs/accession numbers/DOIs will be available only after acceptance of the manuscript for publication so that we can ensure their inclusion before publication.”   The data to be included comprises:   - information in the docx supplementary files/end-matter (the bulk of the material) - information in the file review_data.xlsx   The data has now been reviewed with checks particularly for copyright issues, and is now available, and referred to directly in the text. The DOI has been reserved: <https://10.6084/m9.figshare.26013130>  But until publication, this URL should provide access: <https://figshare.com/s/295fd178b30c7c19773c>  We would welcome advice from the editor regarding whether we should add supplements to the data share as well as keeping them linked to the manuscript itself (i.e., as supplements), or leave figshare solely for the data supplement. |
| 5 | E | Please upload a copy of Figure 2, to which you refer in your text on page 38. If the figure is no longer to be included as part of the submission please remove all reference to it within the text. | Figure 2 refers to the entire 7.6 PRISMA-Flow diagram provided above it, including the explanatory text (directly below the Flow diagram).  Table 5 is now referred to directly next to the table itself, and the footer to the table is clarified. In both cases, we have also now added these as supplements, in their own separate files (they are thus re-numbered).  We had already included modified PRISMA Flow diagrams, and an appropriate PRISMA checklist in the submission (Supplement 5, and Figure 1, with additional detail provided in Supplement 6). The manuscript *is* not a systematic review or meta-analysis, it includes an element that comprises a scoping review, and we have used the appropriate checklist to reflect that.  We have now moved the supplements, and figures, into separate files. To do this we:   1. Split each supplement or figure into its own file, using S1_File.xxx and S1_Fig.xxx filename convention 2. Updated cross-references within the manuscript, ensuring all figures and supplements were referred to inline, and added the list to a final subheading ‘Supporting Information’. 3. Tidied the supplements, to ensure they stand alone, removing footnotes to a ‘context’ heading, and adding references where they appear within the supplement. (We assume that in production the supplements will also be labelled with their origin and license).   Tables that are not supplementary/supporting information remain inline, which we believe is consistent with the instructions to authors. |
| 6 | E | Please ensure that you refer to Figure 2 and 3 in your text as, if accepted, production will need this reference to link the reader to the figure |  |
| 7 | E | We notice that your supplementary [figures/tables] are included in the manuscript file. Please remove them and upload them with the file type 'Supporting Information'. Please ensure that each Supporting Information file has a legend listed in the manuscript after the references list. |  |
| 8 | E | We note that this manuscript is a systematic review or meta-analysis; our author guidelines therefore require that you use PRISMA guidance to help improve reporting quality of this type of study. Please upload copies of the completed PRISMA checklist as Supporting Information with a file name “PRISMA checklist”. |  |
| 9 | E | We note you have included a table to which you do not refer in the text of your manuscript. Please ensure that you refer to Table 5 in your text; if accepted, production will need this reference to link the reader to the Table. |  |
| 10 | 1 | The title does not align with the paper. I was expecting to see some published research heuristics, like work published by Abel Brodeur and others, but that wasn’t there and so I think the paper could be simply retitled to better address the actual content. | We are not familiar with the research heuristics work highlighted here, and although we searched the works on that author’s website we could not find that phrase used. We are aware of methodologically focused work regarding e.g., publication and review standards for reporting of statistics (including regarding p-hacking), although this is of course a related but different topic our own. That poses a challenge for us in understanding the particular mismatch observed, and responding to it. We are happy to consider alternative titles, although we note that our responses to other points regarding the framing of the article may have the indirect effect of responding to this point too; we have made some edits to the title which we hope support this framing. |
| 11 | 1 | The paper gets at an idea which is an important one: we are often missing clear guidelines with respect to ethical behavior in new technologies. AI has scrambled the academic world from top to bottom and we are all struggling to catch up. Understanding a new set of guidelines is essential. Unfortunately, the AI perspective of this feels completely missing or tacked on later to the paper. AI seems to be an addition, rather than the focus. I wonder if the framing could be revised so that AI is a single element, rather than pitched as the focus. Or could it be removed and added as a dynamic considered, just as a mentioned topic. Just reading as is, things feel unbalanced and undelivered, and so more detail is needed, or other reframing is required to better align the stated contribution with the actual research. This comment is the most significant in requiring revision before the paper could be published. | We appreciate the reviewer’s agreement that the paper is directed at an important issue. We have carefully considered how to address the concern regarding framing. We have found it challenging to do so, because although we realise the reviewer is reflecting that this is not the case, in re-reviewing the introduction we believe we have been quite clear that AI is indeed being pointed to as “a single element, rather than pitched as the focus”. That is in two senses, (1) first, in terms of considering existing policy and novel policies that have sought to address emerging technologies, we took a broad search approach, in which AI is the most recent example (and perhaps the one that has triggered most responses, including the proposals outlined in the background Table); and (2) in terms of options or policy development, our realisation was that many of the ways in which issues particular to AI might be addressed, are in fact more general than that, and thus AI provides an example from which sometimes specific issues arise, while the policy approach and its focus on learning is broader than AI. Moreover, of course any novel policy must be situated in the wider policy context, most notably the Decl of Helsinki. That of course has the benefit of also being more responsive to future changes (and preceding challenges).  We have reviewed the whole manuscript and sought to explicitly refer to emerging technologies, and particular policies that have emerged particularly related to emerging technology and AI (we recognise that these may not be obvious, this was an omission on our part).  In the supplements, we do make clear that we *exemplify* the policies with respect to the specific field (education). In the newly formatted supplements this is made clearer. |
| 12 | 2 | At the beginning the article foregrounds AI. I couldn't tell at that point if AI was to be taken as an example of an emerging technology that challenges research ethics in ways of concern in publication or if, more than a passing reference, AI was to the focus of the article and your methods. AI is not mentioned again until the end. It doesn't appear that concerns about AI in particular shaped the methodology, unless as a non-methodologist I have missed something. **Therefore as a reader I would have found it helpful to have more clarity about AI as one among other emerging technologies, perhaps the most pressing on at the moment, that your approach would hope could be addressed by all the parties responsible for ethical research practices in published work.** This appears to be merely a framing problem that should be easily resolved. |  |
| 13 | 1 | Something that I inherently do not understand, with respect to editorial polices and research is: why can an executive decision not be made, and changes implemented? If a journal sets a new policy that X action or Y requirement must be implemented for acceptance, perhaps with a date in the future for implementation, why is this not an option? Some of this article feels fussy around the edge, without letting us understand, as readers, why editors cannot just define new, better, and perhaps more strict ethical criteria. I have long assumed it is inertia, so it would be useful to motivate why things are not simply changed. | Journals sit in part of a wider ecosystem, as noted start of 1.3 and 1.2, and so changes to journal policy are just one part of a dynamic system. This system includes COPE and journal publishers. Organisational learning and change is challenging, and as the data we (and others) report indicates, existing approaches – largely compliance oriented – do not appear to have achieved universal adoption in policy or practice. Most significant for our argument, there are gaps in reporting that make it hard to understand how ethical issues are navigated. The proposals we draw on are grounded in three recent reviews of policy options, and while certainly there is merit to the idea of simply introducing changes with an established timeline, we believe that is most likely to succeed where there is widespread buyin, supported by effective communication and education work. |
| 14 | 1 | The conversation about de facto versus de jure use of IRB was very interesting. I appreciate the perspective that IRBs and similar review boards have been the focus of journals, although we all generally acknowledge that they are insufficient. I would have liked to see a bit more of a discussion of whether these requirements have achieved anything or if we should move away from them. | We think this is largely out of scope for the paper, except insofar as the empirical evidence provides more support for the concern that existing requirements are often not enshrined in journal policies nor adhered to by authors. Section 1.2.2 indeed does discuss some of these concerns, and provides some rationale for the approach taken in the remainder of the manuscript, i.e., that existing approaches (mandates) would be augmented by better foregrounding non-compliance oriented framings of research ethics. We would suggest that journals should adopt the guidelines we produce, but this is likely to supplement rather than replace existing models. |
| 15 | 1 | I want to express some pushback on the recommendation for international IRB – or the perception that there should be a lack of it (on pages 3 and 4). It strikes me as close to soft bigotry. Many international organizations across the globe have excellent IRBs and many countries now have national offices. This recommendation feels very out of line with the current state of IRBs.  o The two references cited 17 and 18 are from 2002 and 2015. Things have significantly changed with respect to IRBs outside of “Western” contexts. The authors should either engage with this with respect to more recent publications; better contextualize that this comment is a perception of editors; and/or remove it. | We appreciate the concern raised here. We reviewed this section within the manuscript. In the penultimate sentence we note that there is international variation in IRB systems that is likely to have implications for whether a project undergoes IRB/REC review. In the final sentence we note a particular justice concern this raises, that requirements for IRB may impact resource poor settings more, perpetuating injustice. We do not believe we make a recommendation (“for international IRB”), and are surprised the language here is construed as making one. We further reviewed the supplements, and similarly make no recommendations regarding IRBs.  We don’t indicate there should be an international IRB (or, conversely, that we should not have broadly agreed standards internationally), but rather provide the factual statement – there is variation internationally. This variation exists across relatively higher and lower resourced countries, and indeed since ~2014 there has been significant change particularly across European countries; we have added some further references that discuss this variation. We have highlighted the particular risk of perpetuating injustice where requirements are imposed that may disproportionately impact lower resourced countries, which certainly isn’t to dismiss the work done already in those contexts, nor to suggest different contexts should have different standards for levels ethics (which also raises risks of ethics dumping). We would also recognise that there is significant discourse re: different standards qua principles and models of application for RECs, i.e., that different models may operationalise differently, and indeed articulate principles in varied ways, while seeking similar ‘standards’ of ethics. We have added some further references here discussing more recent comparisons in different global contexts, and other issues. We have also rephrased that last sentence to highlight a wider range of risks noted in the literature.  International organisations and country national offices, and indeed national and local organisations themselves, may have excellent systems, and these exist particularly in health and development contexts. However, this kind of adoption is somewhat different from either national requirements, or just widespread custom and practice, of IRB-like models in research organisations (including universities).  The paper itself provides an example. It went through a REC process in Australia, ratified in the UK, as a form of Delphi. In the US this might have been considered IRB exempt (which would still result in a paperwork trail and number, although minimal oversight), but in many countries the work would not fall within scope for REC (and it might be a poor use of resources if it did); it is reasonable for journals to expect this to be explained, but on issues like this it is not reasonable to impose one particular model on researchers from varied systems. |
| 16 | 1 | One of the strategy foci presented in what I believe to be Table 5 on page 29 is training. Is this a realistic recommendation. Several of the foci in Table 5 gave me similar pause, but this recommendation stood out. Given the extreme constraints facing journals and editors, is training an actually realistic recommendation? | Those recommendations arise from three recent reviews (as noted in the preamble to the table), and are consolidated here, used in our analysis of policies identified.  We believe these are realistic recommendations, and note that the method for the paper draws on collective expertise of editors (who are also authors) who have standing to make that judgement. This is particularly true for learned society journals (where editorship is likely to be in a wider context of society participation, indeed we have run training or workshops regarding publication), but it is also relevant for publishers, and indeed most publishers to provide learning material to authors/reviews/editors (often in tandem with COPE), to a greater or lesser degree (e.g., at the upper end, some run courses for prospective authors including ECRs and research students). We have elaborated on some of the examples here. Journals are part of the wider research ecosystem, and of course most of their stakeholders have roles outside of specific journal functions (i.e., as researchers), training has cost, but also benefits that have benefits for research quality and (hopefully) also venue processes.  Note that this table is both incorporated into the paper, as background literature, and into the policy material outputs (supplements, Table 5 – now excerpted). |
| 17 | 1 | It would be useful to understand what defines social science. At present a lot of it seems to simply be “education”. Which is fine but is not encompassing of social science. If this is the case (e.g., that the focus is on education not necessarily social science), the entire paper should be reframed. | We believe this comment may partly be directed at the supplements (in which Table 5, comment #16, also appears), which is where there is a more significant attention to learning and technologies research. As we explain in the introduction, we use the specific domain as a way to exemplify some issues, and the application of the guidelines developed to a specific, in a way we hope other fields will find useful in supporting their authors/reviewers/readers to engage with the issues in their particular contexts.  As noted *“The present paper bridges this gap through systematic analysis of existing editorial policy, and development of new policies targeting development of ethical sensitivity. While we draw on broad disciplinary context via a scoping review, we recognise that the inherent diversity within different scholarly domains requires tailoring to specific areas. Therefore, this paper provides exemplification through the discipline of ‘learning and technology’, targeting emerging issues regarding research ethics and AI applications in education. Our approach thus draws out general principles, while acknowledging the inherent diversity across disciplines, and their distinct challenges.*”, with section 1.2.2 setting out the challenge as cross-disciplinary, section 2.1 setting out our cross-disciplinary search strategy, and section 3.2 providing results of our cross-disciplinary analysis, and section 3.2 a set of journal policies (these are in fact cross-disciplinary, although the publishers were sampled from educational technology journals). The one reference to social science (in the abstract) has been removed, and we have made minor edits in the paper and manuscript to clarify scope. |
| 18 | 1 | Ultimately, I read this paper twice through my process of reading and reviewing. On my second reading, I came away with the impression that it was several disjointed sections adhered together. This, of course, is not necessarily relevant to PLOS ONE, which wants to publish unique and well-done work – which I do think this paper is. But, if this is to be a useful piece for anyone but the authors, significant revisions are needed to make it coherent, comprehensible, and actionable. | We have sought to improve the flow and framing of the paper throughout, while aiming for brevity; we of course also recognise that some of the challenges presented in the introduction are relevant to a range of approaches, and the method and outputs adopted necessarily focus on a particular component of the ethics ecosystem. |

# Decision Letter

PONE-D-24-01120

How do we learn about research ethics from published research? Developing editorial policy for emerging technologies using a scoping review and reference panel

PLOS ONE


Thank you for submitting your manuscript to PLOS ONE. After careful consideration, we feel that it has merit but does not fully meet PLOS ONE’s publication criteria as it currently stands. Therefore, we invite you to submit a revised version of the manuscript that addresses the points raised during the review process.

Please submit your revised manuscript by Jul 25 2024 11:59PM. If you will need more time than this to complete your revisions, please reply to this message or contact the journal office at [plosone@plos.org](mailto:plosone@plos.org). When you're ready to submit your revision, log on to <https://www.editorialmanager.com/pone/> and select the 'Submissions Needing Revision' folder to locate your manuscript file.

Please include the following items when submitting your revised manuscript:

- A rebuttal letter that responds to each point raised by the academic editor and reviewer(s). You should upload this letter as a separate file labeled 'Response to Reviewers'.
- A marked-up copy of your manuscript that highlights changes made to the original version. You should upload this as a separate file labeled 'Revised Manuscript with Track Changes'.
- An unmarked version of your revised paper without tracked changes. You should upload this as a separate file labeled 'Manuscript'.

If you would like to make changes to your financial disclosure, please include your updated statement in your cover letter. Guidelines for resubmitting your figure files are available below the reviewer comments at the end of this letter.

If applicable, we recommend that you deposit your laboratory protocols in [protocols.io](http://protocols.io) to enhance the reproducibility of your results. Protocols.io assigns your protocol its own identifier (DOI) so that it can be cited independently in the future. For instructions see: <https://journals.plos.org/plosone/s/submission-guidelines#loc-laboratory-protocols>. Additionally, PLOS ONE offers an option for publishing peer-reviewed Lab Protocol articles, which describe protocols hosted on [protocols.io](http://protocols.io). Read more information on sharing protocols at <https://plos.org/protocols?utm_medium=editorial-email&utm_source=authorletters&utm_campaign=protocols>.

We look forward to receiving your revised manuscript.

Kind regards,

Primus Che Chi, Ph.D
Academic Editor
PLOS ONE

Journal Requirements:

When submitting your revision, we need you to address these additional requirements.

1. Please ensure that your manuscript meets PLOS ONE's style requirements, including those for file naming. The PLOS ONE style templates can be found at 
<https://journals.plos.org/plosone/s/file?id=wjVg/PLOSOne_formatting_sample_main_body.pdf> and 
<https://journals.plos.org/plosone/s/file?id=ba62/PLOSOne_formatting_sample_title_authors_affiliations.pdf>

2. Thank you for stating the following in the Competing Interests section: 
"The research reported was instigated as a collaboration between the Journal of Learning Analytics (JLA), Australasian Journal of Educational Technology (AJET), and the British Journal of Educational Technology (BJET), by the lead author, who was a previous co-editor-in-chief of the JLA. The authors acknowledge BJET co-editor-in-chief Cathy Lewin, who joined the journal later in this process, and thus did not participate in authoring this piece.
The views expressed are those of the authors, and may not represent the views of the journals, scholarly societies, or other organisations to which they are affiliated. 
The work was begun over the course of the lead author’s sabbatical, which included periods co-located at UCL and KTH / the Swedish Digital Futures research centre, supporting direct interaction with editors based at those institutions."

Please confirm that this does not alter your adherence to all PLOS ONE policies on sharing data and materials, by including the following statement: ""This does not alter our adherence to  PLOS ONE policies on sharing data and materials.” (as detailed online in our guide for authors <http://journals.plos.org/plosone/s/competing-interests>).  If there are restrictions on sharing of data and/or materials, please state these. Please note that we cannot proceed with consideration of your article until this information has been declared.

Please include your updated Competing Interests statement in your cover letter; we will change the online submission form on your behalf.

3. Please note that in order to use the direct billing option the corresponding author must be affiliated with the chosen institute. Please either amend your manuscript to change the affiliation or corresponding author, or email us at [plosone@plos.org](mailto:plosone@plos.org) with a request to remove this option.

4. When completing the data availability statement of the submission form, you indicated that you will make your data available on acceptance. We strongly recommend all authors decide on a data sharing plan before acceptance, as the process can be lengthy and hold up publication timelines. Please note that, though access restrictions are acceptable now, your entire data will need to be made freely accessible if your manuscript is accepted for publication. This policy applies to all data except where public deposition would breach compliance with the protocol approved by your research ethics board. If you are unable to adhere to our open data policy, please kindly revise your statement to explain your reasoning and we will seek the editor's input on an exemption. Please be assured that, once you have provided your new statement, the assessment of your exemption will not hold up the peer review process.

5. Please upload a copy of Figure 2, to which you refer in your text on page 38. If the figure is no longer to be included as part of the submission please remove all reference to it within the text.

6. Please ensure that you refer to Figure 2 and 3 in your text as, if accepted, production will need this reference to link the reader to the figure.

7. We note you have included a table to which you do not refer in the text of your manuscript. Please ensure that you refer to Table 5 in your text; if accepted, production will need this reference to link the reader to the Table.

8. We notice that your supplementary [figures/tables] are included in the manuscript file. Please remove them and upload them with the file type 'Supporting Information'. Please ensure that each Supporting Information file has a legend listed in the manuscript after the references list.

9. We note that this manuscript is a systematic review or meta-analysis; our author guidelines therefore require that you use PRISMA guidance to help improve reporting quality of this type of study. Please upload copies of the completed PRISMA checklist as Supporting Information with a file name “PRISMA checklist”.

**Additional Editor Comments:**

Dear Authors,
We have received some detailed and excellent feedback from two reviewers. The reviewers have raised substantial issues with the manuscripts and how these issued can be addressed in the next revision of the manuscript. The most important of the issues raised are the role of AI technology within the manuscript and coherence/connectedness of the various sections of the write-up. I am confident that if these issues are carefully reviewed and addressed by the authors it would strengthen the paper and improve the chances of being recommended for publication.

Best wishes!


[Note: HTML markup is below. Please do not edit.]

Reviewers' comments:

Reviewer's Responses to Questions

**Comments to the Author**

1. Is the manuscript technically sound, and do the data support the conclusions?

The manuscript must describe a technically sound piece of scientific research with data that supports the conclusions. Experiments must have been conducted rigorously, with appropriate controls, replication, and sample sizes. The conclusions must be drawn appropriately based on the data presented.

Reviewer #1: Partly

Reviewer #2: Yes

2. Has the statistical analysis been performed appropriately and rigorously?

Reviewer #1: N/A

Reviewer #2: I Don't Know

3. Have the authors made all data underlying the findings in their manuscript fully available?

The [PLOS Data policy](http://www.plosone.org/static/policies.action#sharing) requires authors to make all data underlying the findings described in their manuscript fully available without restriction, with rare exception (please refer to the Data Availability Statement in the manuscript PDF file). The data should be provided as part of the manuscript or its supporting information, or deposited to a public repository. For example, in addition to summary statistics, the data points behind means, medians and variance measures should be available. If there are restrictions on publicly sharing data—e.g. participant privacy or use of data from a third party—those must be specified.

Reviewer #1: Yes

Reviewer #2: Yes

4. Is the manuscript presented in an intelligible fashion and written in standard English?

PLOS ONE does not copyedit accepted manuscripts, so the language in submitted articles must be clear, correct, and unambiguous. Any typographical or grammatical errors should be corrected at revision, so please note any specific errors here.

Reviewer #1: Yes

Reviewer #2: Yes

5. Review Comments to the Author

Please use the space provided to explain your answers to the questions above. You may also include additional comments for the author, including concerns about dual publication, research ethics, or publication ethics. (Please upload your review as an attachment if it exceeds 20,000 characters)

Reviewer #1: Thank you for the opportunity to review “How do we learn about research ethics from published research? Developing editorial policy for emerging technologies using a scoping review and reference panel”. The paper seeks to develop a new editorial policy for research ethics across social science. The paper reports original research, not published elsewhere, though requires additional detail to sufficiently describe the research and resulting conclusions.

My comments focus on areas in which to provide more information to better align the stated goals of the paper with the actual achievements of the paper, and to sufficiently describe the research so as to understand what is done:

• The title does not align with the paper. I was expecting to see some published research heuristics, like work published by Abel Brodeur and others, but that wasn’t there and so I think the paper could be simply retitled to better address the actual content.

• The paper gets at an idea which is an important one: we are often missing clear guidelines with respect to ethical behavior in new technologies. AI has scrambled the academic world from top to bottom and we are all struggling to catch up. Understanding a new set of guidelines is essential. Unfortunately, the AI perspective of this feels completely missing or tacked on later to the paper. AI seems to be an addition, rather than the focus. I wonder if the framing could be revised so that AI is a single element, rather than pitched as the focus. Or could it be removed and added as a dynamic considered, just as a mentioned topic. Just reading as is, things feel unbalanced and undelivered, and so more detail is needed, or other reframing is required to better align the stated contribution with the actual research. This comment is the most significant in requiring revision before the paper could be published.

• Something that I inherently do not understand, with respect to editorial polices and research is: why can an executive decision not be made, and changes implemented? If a journal sets a new policy that X action or Y requirement must be implemented for acceptance, perhaps with a date in the future for implementation, why is this not an option? Some of this article feels fussy around the edge, without letting us understand, as readers, why editors cannot just define new, better, and perhaps more strict ethical criteria. I have long assumed it is inertia, so it would be useful to motivate why things are not simply changed.

• The conversation about de facto versus de jure use of IRB was very interesting. I appreciate the perspective that IRBs and similar review boards have been the focus of journals, although we all generally acknowledge that they are insufficient. I would have liked to see a bit more of a discussion of whether these requirements have achieved anything or if we should move away from them.

• I want to express some pushback on the recommendation for international IRB – or the perception that there should be a lack of it (on pages 3 and 4). It strikes me as close to soft bigotry. Many international organizations across the globe have excellent IRBs and many countries now have national offices. This recommendation feels very out of line with the current state of IRBs.
o The two references cited 17 and 18 are from 2002 and 2015. Things have significantly changed with respect to IRBs outside of “Western” contexts. The authors should either engage with this with respect to more recent publications; better contextualize that this comment is a perception of editors; and/or remove it.

• One of the strategy foci presented in what I believe to be Table 5 on page 29 is training. Is this a realistic recommendation. Several of the foci in Table 5 gave me similar pause, but this recommendation stood out. Given the extreme constraints facing journals and editors, is training an actually realistic recommendation?

• It would be useful to understand what defines social science. At present a lot of it seems to simply be “education”. Which is fine but is not encompassing of social science. If this is the case (e.g., that the focus is on education not necessarily social science), the entire paper should be reframed.

• Ultimately, I read this paper twice through my process of reading and reviewing. On my second reading, I came away with the impression that it was several disjointed sections adhered together. This, of course, is not necessarily relevant to PLOS ONE, which wants to publish unique and well-done work – which I do think this paper is. But, if this is to be a useful piece for anyone but the authors, significant revisions are needed to make it coherent, comprehensible, and actionable.

Reviewer #2: At the beginning the article foregrounds AI. I couldn't tell at that point if AI was to be taken as an example of an emerging technology that challenges research ethics in ways of concern in publication or if, more than a passing reference, AI was to the focus of the article and your methods. AI is not mentioned again until the end. It doesn't appear that concerns about AI in particular shaped the methodology, unless as a non-methodologist I have missed something. Therefore as a reader I would have found it helpful to have more clarity about AI as one among other emerging technologies, perhaps the most pressing on at the moment, that your approach would hope could be addressed by all the parties responsible for ethical research practices in published work. This appears to be merely a framing problem that should be easily resolved.

6. PLOS authors have the option to publish the peer review history of their article ([what does this mean?](https://journals.plos.org/plosone/s/editorial-and-peer-review-process#loc-peer-review-history)). If published, this will include your full peer review and any attached files.


If you choose “no”, your identity will remain anonymous but your review may still be made public.


**Do you want your identity to be public for this peer review?** For information about this choice, including consent withdrawal, please see our [Privacy Policy](https://www.plos.org/privacy-policy).

Reviewer #1: No

Reviewer #2: **Yes:** Jonathan D Moreno

[NOTE: If reviewer comments were submitted as an attachment file, they will be attached to this email and accessible via the submission site. Please log into your account, locate the manuscript record, and check for the action link "View Attachments". If this link does not appear, there are no attachment files.]

While revising your submission, please upload your figure files to the Preflight Analysis and Conversion Engine (PACE) digital diagnostic tool, <https://pacev2.apexcovantage.com/>. PACE helps ensure that figures meet PLOS requirements. To use PACE, you must first register as a user. Registration is free. Then, login and navigate to the UPLOAD tab, where you will find detailed instructions on how to use the tool. If you encounter any issues or have any questions when using PACE, please email PLOS at [figures@plos.org](mailto:figures@plos.org). Please note that Supporting Information files do not need this step.
